# Atomistic Simulations of the Permeability and Dynamic Transportation Characteristics of Diamond Nanochannels

**DOI:** 10.3390/nano12111785

**Published:** 2022-05-24

**Authors:** Bingqing Li, Bin Dong, Tianxiang Shi, Haifei Zhan, Yongqiang Zhang

**Affiliations:** 1 College of Civil Engineering and Architecture, Zhejiang University, Hangzhou 310058, China; 21912097@zju.edu.cn (B.L.); 22012088@zju.edu.cn (B.D.); stxzj@zju.edu.cn (T.S.); 2 School of Mechanical, Medical and Process Engineering, Queensland University of Technology (QUT), Brisbane, QLD 4001, Australia; 3 Center for Materials Science, Queensland University of Technology (QUT), Brisbane, QLD 4001, Australia

**Keywords:** diamond nanochannel, permeability, flow rate, transition nominal pressure, molecular dynamics simulation

## Abstract

Through atomistic simulations, this work investigated the permeability of hexagonal diamond nanochannels for NaCl solution. Compared with the multilayer graphene nanochannel (with a nominal channel height of 6.8 Å), the diamond nanochannel exhibited better permeability. The whole transportation process can be divided into three stages: the diffusion stage, the transition stage and the flow stage. Increasing the channel height reduced the transition nominal pressure that distinguishes the diffusion and flow stages, and improved water permeability (with increased water flux but reduced ion retention rate). In comparison, channel length and solution concentration exerted ignorable influence on water permeability of the channel. Further simulations revealed that temperature between 300 and 350 K remarkably increased water permeability, accompanied by continuously decreasing transition nominal pressure. Additional investigations showed that the permeability of the nanochannel could be effectively tailored by surface functionalization. This work provides a comprehensive atomic insight into the transportation process of NaCl solution in a diamond nanochannel, and the established understanding could be beneficial for the design of advanced nanofluidic devices.

## 1. Introduction

Inspired by biological nanochannels, synthetic nanochannels have attracted increasing interest due to their unique transmission behaviors and promising prospects in various fields, such as biomedicine [1,2,3], nanofiltration [4,5], water desalination [6,7,8,9], and energy storage [10,11,12]. The hydration interactions, van der Waal interactions, and electrostatic interactions are the main factors that confine the transition behaviors of aqueous solutions down to nanoscale [13,14,15,16]. Extensive efforts have been devoted to investigating the characteristics of nanofluids based on experiments [17,18,19,20,21] or theoretical analysis [22,23,24], covering different types of synthetic nanochannels [24], such as carbon nanotubes (CNTs) [25,26,27,28,29,30,31], nanopores [32,33,34,35], and functionalized nanopores [36,37,38,39,40,41]. Due to their atomically-smooth hydrophobic graphitic surface and nanoscale confinements, sp2 carbon-based nanochannels are the most studied nanofluidic structure. It has been found that pressure direction can be tuned to optimize permeability for ionic transport through a CNT [30], and several orders of magnitude of higher flow rate inside CNTs have been reported.

Due to structural similarity, fluid transportation characteristics within two-dimensional (2D) graphene slits [14,42,43,44,45,46] have also been extensively discussed. The interlayer distance, or the channel height, determines the degree of confinement on the solution, and a wide range of factors affect the transportation behavior of the confined solutions, such as atomic structure, surface curvature, surface charge [18], chirality [47], pressure [48], and in-plane strain [49]. Due to its low bending stiffness, graphene is often presented with strong wrinkles, which could introduce strong impact on the solution behavior in nanofluidic devices [50]. Recently, researchers successfully prepared ultrathin 2D diamond [51,52,53], which provides a new candidate to construct 2D nanochannels. Theoretical works reveal that 2D diamond inherits the excellent mechanical properties of bulk diamond, and maintains a very high bending rigidity, compared with that of graphene [54].

To this end, this work aims to explore the transportation characteristics of sodium chloride solution in two-dimensional diamond channels using molecular dynamics simulation. The transportation behaviors of sodium chloride solution in both graphene and diamond nanochannels are investigated, and the influences from various factors, including channel length, channel height, temperature, concentration of the NaCl solution and surface functionalization, are discussed. This work provides a theoretical basis and design reference for the application of diamond nanochannels in desalination, which may be beneficial for the design of new carbon-based nanofluidic devices.

## 2. Methods

**Figure 1:**
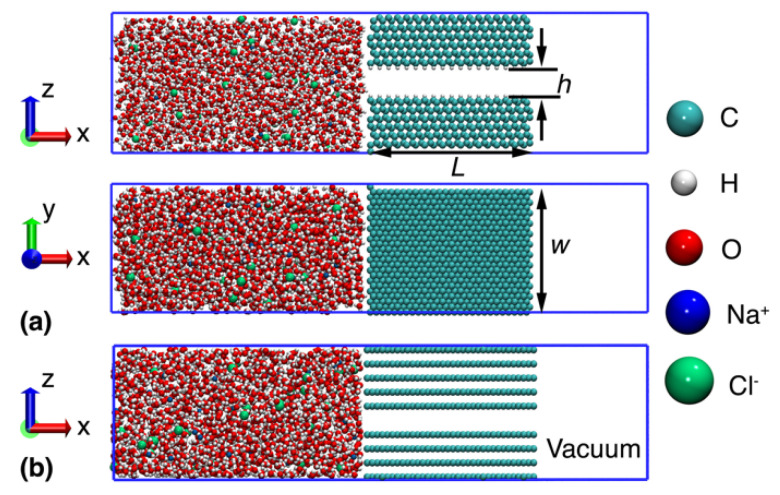
Schematical view of the simulation model. (**a**) Nanochannel constructed from nanometer-thick diamond. *L*, *h* and w representing the channel length, height, and width, respectively; and (**b**) Nanochannel constructed from multilayer graphene.

The nanochannel was constructed from two layers of hexagonal diamond—lonsdaleite. The two free surfaces of the channel were passivated by H atoms, which created a diamane like structure [53]. Each layer had a length of about 4 nm (*x*-axis) and a width of about 3 nm (*y*-axis). As illustrated in Figure 1a, the initially considered nanochannel had a height of about 6.8 Å, and the diamond layer had a height of about 30 Å. Note that the channel height represents the nominal distance between the H atoms of the upper and lower layers, and the effective distance was about 4.4 Å, considering the van der Waal radius of H as 1.2 Å. A reservoir was created on the left of the nanochannel with a length of 60 Å, which was filled with 1M sodium chloride aqueous solution. Meanwhile, a vacuum space was created on the right side of the nanochannel for the transportation simulation. After that, water molecules, sodium and chloride ions were packed into the reservoir using the open-source package Packmol [55]. The overall size of the simulation box was about 130 × 30 × 30 Å^3^. Periodic boundary conditions were adopted in the width and height directions (*y*- and *z*-axis) during the simulation. For comparison purposes, a multilayer graphene-based nanochannel [56], with a similar geometrical parameter, was also constructed (Figure 1b), i.e., the graphene has nine layers, with a length and width of about 40 × 30 Å^2^. The channel height is kept the same as the diamond nanochannel at 6.8 Å, i.e., the distance between carbon atoms in the upper and bottom multilayer graphene (with an effective distance of 3.4 Å, considering the van der Waal radius of C as 1.7 Å).

The atomic interactions within and between water molecules were described by the simple point charge expansion model (SPC/E) [57]. The commonly used AIREBO [58] potential was adopted to describe the atomic interactions within the diamond channel, including C-C, C-H, and H-H interactions. Atomic interactions within NaCl, and the atomic interactions between water molecules, NaCl and the nanochannel were treated by the Lennard-Jones (LJ) potential using the Lorentz-Berthelot combination rule. The LJ potential was expressed as,

(1) $$U\left(r\right)=4\varepsilon \left[{\left(\frac{\sigma }{r}\right)}^{12}-{\left(\frac{\sigma }{r}\right)}^{6}\right]$$

where r is the distance between pairs of atoms; ε reflects the depth of the potential energy curve, while the magnitude of σ represents the equilibrium distance between atoms. The corresponding parameters [42] are listed in Supplementary Information Table S1, with a uniform cut-off distance of 12 Å.

All simulations were performed by the open-source package LAMMPS [59]. The system was first relaxed to the energy minimum status and then equilibrated at 300 K and 1 atm for 50 ps under the isothermal isobaric ensemble (NPT). Afterwards, a virtual wall was applied on the left of the water reservoir to push the solution forward at a constant velocity of 0.1 Å/ps. The virtual wall exerts a repulsive force on adjacent atoms expressed by $F=-k{\left(r-R\right)}^{2}$, where *k* = 1 is a constant, *R* is the position of the virtual wall and *r*-*R* is the distance from the atom to the virtual wall. To mitigate the influence from pressure fluctuation, the whole system was relaxed for 5 ps under the microcanonical ensemble (NVE) for every 0.2 Å virtual wall displacement. The reaction force on the virtual wall was averaged from the last two ps relaxation results. The simulation ceased when the reservoir was reduced to one third of its original volume. The nanochannels were fixed rigid during the simulation. A time step of 0.5 fs was applied for all simulations.

## 3. Results and Discussion

### 3.1. Transportation Characteristics in a Nanochannel

**Figure 2:**
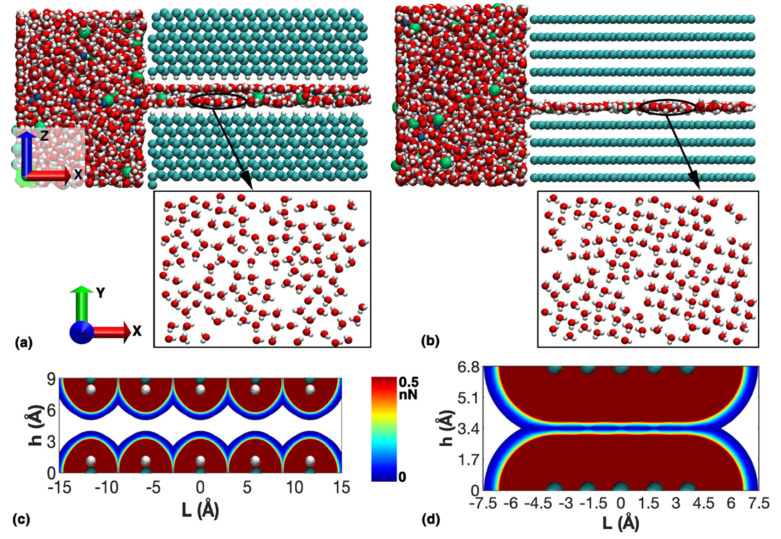
NaCl solution transportation within the diamond and multilayer graphene nanochannel. (**a**) The NaCl solution in the diamond nanochannel (**upper** panels) and the structure of the water molecules (**lower** panel); (**b**) The NaCl solution in the multilayer graphene nanochannel (**upper** panel) and the structure of water molecules (**lower** panel); and LJ energy domain within: (**c**) the diamond nanochannel; and (**d**) the multilayer graphene nanochannel. Here, only the repulsive force less than 0.5 nN is visualized here.

Firstly, we compared the transportation behavior of 1M NaCl solution between the graphene and diamond nanochannels, which shared the same channel height of 6.8 Å. As shown in Figure 2a, the NaCl solution exhibited a bilayer structure in the diamond nanochannel after a total of 1200 ps simulation (see Supplementary Material Video S1 for the transportation process). In comparison, a monolayer solution was shown in the graphene nanochannel (Figure 2b, see Supplementary Material Video S2 for the transportation process). In contrast, the diamond channel exhibits higher permeability (see Supplementary Material Figure S1). According to the atomic configurations, some parts of the water layers exhibited a quasi-square molecular arrangement, either in the diamond or graphene channel. Such layered phenomena originated from the difference of the energy surface within the nanochannel. For the diamond nanochannel, the surface was passivated by H atoms, while the bilayer graphene had a C surface that resulted in stronger energy domain in the nanochannel. Considering the long-range van der Waal (vdW) interaction within the nanochannel, we can qualitatively demonstrate the energy domain by visualization of the LJ interactions between two atomic chains. For simplicity, only the repulsive energy in the channels is illustrated. As plotted in Figure 2c, the diamond surface is represented by a C-H diatomic chain. Strong repulsive fields are formed on each side of the upper and lower diamond layers. Such observation agrees well with the two-layered NaCl solution formed in the nanochannel. In comparison, C atoms in the multilayer graphene nanochannel are closer to each other. The repulsive interactions between the upper and lower diamond layer can be effectively cancelled out in the middle region, and thus form a regime with weak energy domain (Figure 2d). Thus, only a monolayer solution is formed in the narrow middle region where the repulsive field cancelled out.

**Figure 3:**
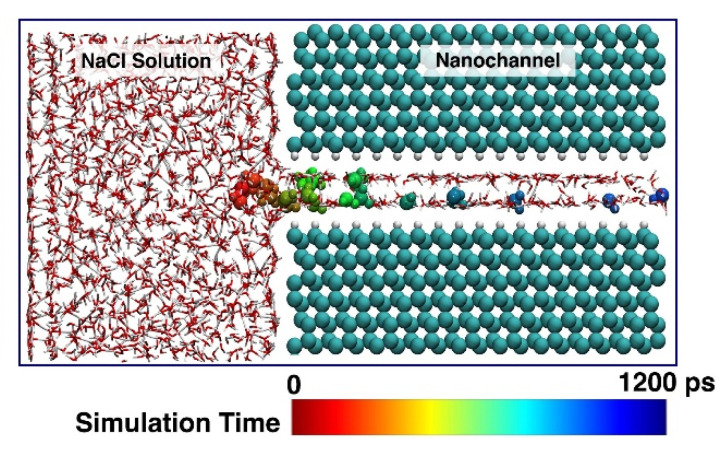
Location trajectory of a selected water molecule in the diamond nanochannel. The location of the water molecule is colored according to the simulation time.

Figure 3 highlights the location trajectory of a randomly selected water molecule during the simulation. As can be seen, the location of the selected water molecule moves around the entry of the nanochannel until the simulation time of ~900 ps. Afterwards, it starts to pass through the channel. The varying locations of the water molecule in the nanochannel indicate that the upper and lower layers of water molecules exchange during the whole simulation.

**Figure 4:**
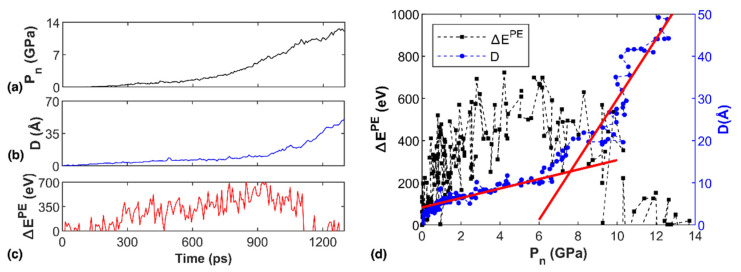
NaCl solution transportation in the diamond nanochannel. (**a**) The pressure trajectory; (**b**) The displacement of the water molecules trajectory; (**c**) The potential energy change trajectory; and (**d**) The potential energy change and displacement as a function of pressure. Two solid linear fitting lines identified the transition nominal pressure.

With the above understanding, we investigated how the properties of the system change during the simulation. According to Figure 4a, the nominal pressure ($P_{n}$) of the reservoir increased continuously due to the moving virtual wall during the transportation simulation, which exhibited a nonlinear relationship with time. Here, the nominal pressure is calculated from $P_{n}=F/A$, where F and A are the reaction force and the area of the virtual wall, respectively. The displacement (D) of the solution showed a similar nonlinear increasing profile (Figure 4b), while the changing gradient of the profile suggested different transportation stages occurred during the simulation. Here, *D* refers to the maximum coordinate of the NaCl solution in the channel relative to the entry of the channel along the transportation direction (*x*-axis). In detail, *D* increased gradually before a simulation time of around 900 ps, corresponding to a displacement less than 10 Å. Afterwards, it increased remarkably with simulation time, indicating much apparent transportation of the NaCl solution in the channel. In comparison, the potential energy change ($∆E^{PE}$) of the system had a totally different profile. Here, $∆E^{PE}=E_{t}^{PE}-E_{0}^{PE}$, with $E_{t}^{PE}$ and $E_{0}^{PE}$ representing the potential energy of the system at simulation time *t* and initial status, respectively. According to Figure 4c, $∆E_{PE}$ increased continuously until a simulation time of about 900 ps, which aligned well with the time when *D* increased significantly. After the threshold value, $∆E^{PE}$ decreased continuously with increasing simulation time, suggesting that the accumulated potential energy was released through the quick transportation of the NaCl solution. Particularly, $∆E^{PE}$ dropped to zero after 1100 ps and fluctuated around zero afterwards. Referring to Figure 4b, it can be seen that the NaCl solution reached the exit of the channel with D around 40 Å at 1200 ps. In other words, the accumulated potential energy was released by the flow transportation of the solution.

Figure 4d further illustrates the relationship between nominal pressure, displacement and potential energy change of the system. It is seen that the relationship between $∆E^{PE}$ (or D) and $P_{n}$ is similar to its relationship with simulation time. Recall the D profile in Figure 4b, the whole simulation can be divided into three stages: the diffusion stage before ~900 ps (only small displacements occurred), the transition stage between 900 and 1100 ps, and the flow stage after 1100 ps. It is estimated that the flow velocity (i.e., the gradient of the displacement profile) in the flow stage was about 0.3 Å/ps, which was more than 30 times faster than that in the diffusion stage (~9.1 × 10^−3^ Å/ps). Since pressure is the driving force for flow transportation, we can thus define a transition nominal pressure ($P_{n}^{t}$) for the diamond nanochannel that determines when flow transportation occurred, similar to the definition of glass transition temperature in polymers [60,61,62,63]. As plotted in Figure 4d, the pressure threshold between the diffusion and flow stages is defined as the transition nominal pressure, which was about 7.7 GPa for the diamond nanochannel. For the multilayer graphene counterpart, the same $P_{n}^{t}$ of 7.7 GPa was estimated. According to Figure 4d, $∆E^{PE}$ reached its maximum magnitude around the transition nominal pressure. After reaching the transition nominal pressure, the pressure exceeded the adhesive constraint from the nanochannel, and thus the solution exhibited flow transportation.

### 3.2. Factors Influencing the Transportation Behavior

Focusing on the diamond nanochannel, we then investigated how different factors affected the transportation behavior of the NaCl solution in the nanochannel, including height, length, temperature and concentration of the NaCl solution. For all simulations, the volume of the NaCl solution reservoir was kept the same. The detailed model information for all models is given in Supplementary Material Table S2.

**Figure 5:**
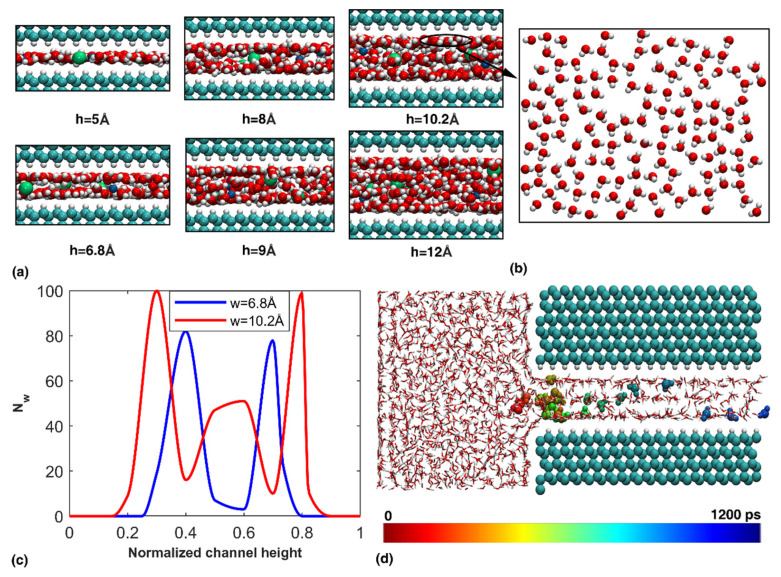
Transportation of NaCl solution in the diamond nanochannel with different heights. (**a**) Atomic configuration of the solution in the channel. Only part of the channel is visualized; (**b**) The water structure inside the channel; (**c**) Distribution of water molecules in the nanochannel; and (**d**) Location trajectory of a selected water molecule during the simulation for a channel height of 10.2 Å.

Different channel heights ranging from 5 to 12 Å were considered first. For comparison purposes, the simulated system had the same size with the same 1M NaCl solution reservoir, and the temperature was kept at 300 K. As compared in Figure 5a, a monolayer of solution was formed when the channel height was reduced to 5 Å, similar to that observed in the graphene nanochannel. With increasing channel height, more solution entered the channel, and molecules in the top and bottom layers exhibited certain square lattices (Figure 5b). The location trajectory of a randomly selected water molecule suggests the upper and lower water layer exchanged molecules during the whole simulation. Due to the formation of water layers, a higher density of water molecules was observed adjacent to the nanochannel boundary, as shown in Figure 5c. Here, the total number of water molecules ($N_{w}$) within the nanochannel were counted along the height direction after the simulation was complete (i.e., after 1300 ps).

**Figure 6:**
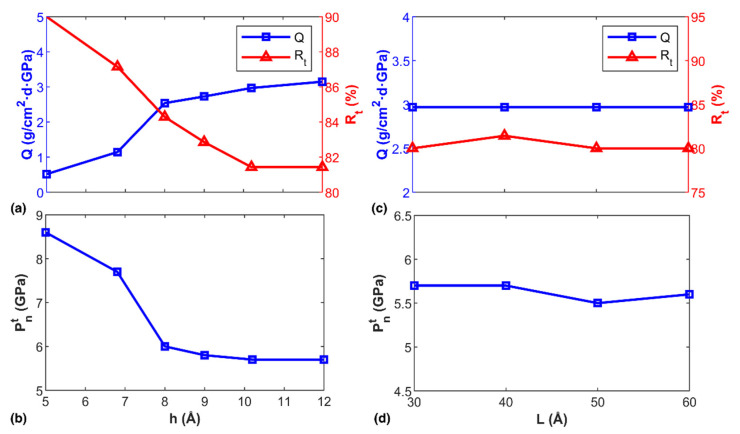
Transportation properties of NaCl solution in diamond channels with different heights and lengths. (**a**) The water flux and ion retention rate as a function of the channel height; and (**b**) The transition pressure as a function of the channel height; (**c**) The water flux and ion retention rate as a function of the channel length; and (**d**) The transition pressure as a function of the channel length.

According to Figure 6a, the water flux increased from 0.515 to 3.148 g/cm^2^·d·GPa when the channel height increased from 5 to 12 Å, suggesting strong dependency of water permeability on channel height (the transportation process for the channel height of 12 Å is shown in Supplementary Material Video S3). Along with increasing water flux (*Q*), the ion retention rate ($R_{t}$) decreased. Specifically, a sharp reduction of $R_{t}$ was observed when the channel height was between 5 and 10 Å. With increasing channel height, $R_{t}$ reached a saturated magnitude above 80%, suggesting excellent ion retention capacity of the diamond channel. All examined samples showed the existence of transition pressure similar to that observed in Figure 4d (see the representative results from the structure with a channel height of 10.2 Å in Supplementary Material Figure S2). As expected, $P_{n}^{t}$ decreased with channel height (Figure 6b). However, when channel height was larger than ~8 Å, $P_{n}^{t}$ became less dependent on channel height, though its magnitude was still as high as 5.8 GPa (for H=12 Å). It was expected that further increasing the channel height would further suppress $P_{n}^{t}$, due to the reduced influence from the channel walls [64,65]. By varying the channel length from 30 to 60 Å (keeping a constant channel height of 10.2 Å), it was found that channel length exerted insignificant impact on water flux and ion retention rate (Figure 6c). Similarly, the transition pressure fluctuated around 5.7 GPa (Figure 6d) when the channel length increased. These results indicate that channel length exerts ignorable influence on the permeability of the diamond channel.

**Figure 7:**
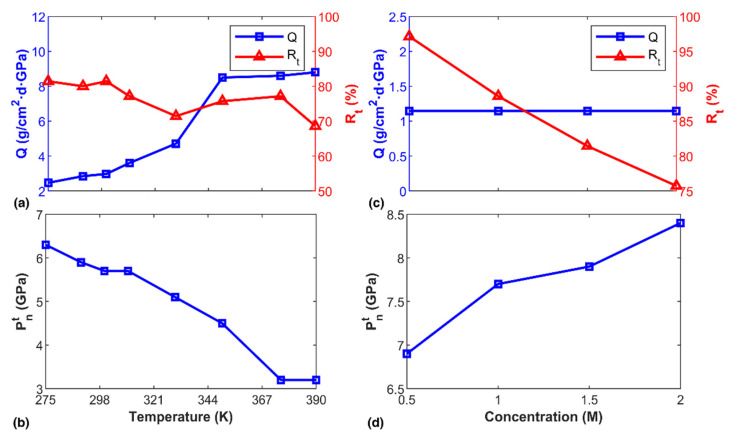
Transportation properties under different temperature and ion concentration. (**a**) The water flux and ion retention rate as a function of temperature; and (**b**) the corresponding transition pressure; (**c**) The water flux and ion retention rate as a function of ion concentration; and (**d**) the corresponding transition pressure.

It is of great interest to probe into how temperature influences transportation behavior, as it determines the kinetic energy of water molecules or ions. For such a purpose, we adopted the diamond nanochannel with a height of 10.2 Å under different temperatures. According to the atomic configurations, layered water molecules were observed in all examined temperatures, and strong exchange of water molecules between the upper and bottom layers was observed (see Supplementary Material Figure S3). As shown in Figure 7a, the water flux increased when temperature increased. Specially, *Q* increased remarkably from 2.969 to 8.6 g/cm^2^·d·GPa when temperature increased from 300 to 350 K. It was found that *Q* saturated to 8.8 g/cm^2^·d·GPa when temperature approached the boiling point of water. In comparison, the ion retention rate fluctuated around 81% when the temperature was less than 300 K, and a significant reduction was observed when temperature rose to 350 K. These observations are reasonable as water molecules possess higher kinetic energy at higher temperature, and thus it is easier for them to get through the nanochannel, which agrees with what was observed in functionalized graphene nanopores [39]. Meanwhile, considering a constant cohesive energy of water (interactions between water molecules), increasing kinetic energy would make it easier for NaCl to flow. As evidenced in Figure 7b, a continuous decrease of the transition temperature was observed, signifying an easier flow stage could be triggered at a higher temperature.

Another factor that influences the permeability and kinematic properties of the nanochannel is the content of salt ions. In the NaCl solution, the ions interact with their surrounding water molecules, and a certain number of water molecules form a hydration shell around the ions. Considering the nanochannel with a height of 6.8 Å (under 300 K), we thus conducted additional simulations by varying ion concentration from 0.5 to 2.0 M. Surprisingly, the ion concentration was found to exert ignorable influence on water flux (Figure 7c), whereas the ion retention rate exhibited a strong linear decreasing relationship with ion concentration. In other words, more ions presented in the solution would promote ion transportation through the diamond nanochannel. It was expected that once NaCl ions entered the nanochannel, they would still interact with water molecules inside the channel and re-attract surrounding water molecules to form a new hydration shell. Such a hydration shell would adversely influence the flow of the solution within the nanochannel. As such, increasing the ion concentration was anticipated to increase the transition pressure, which was affirmed from the simulation, as shown in Figure 7d.

### 3.3. Functionalized Diamond Nanochannel

**Figure 8:**
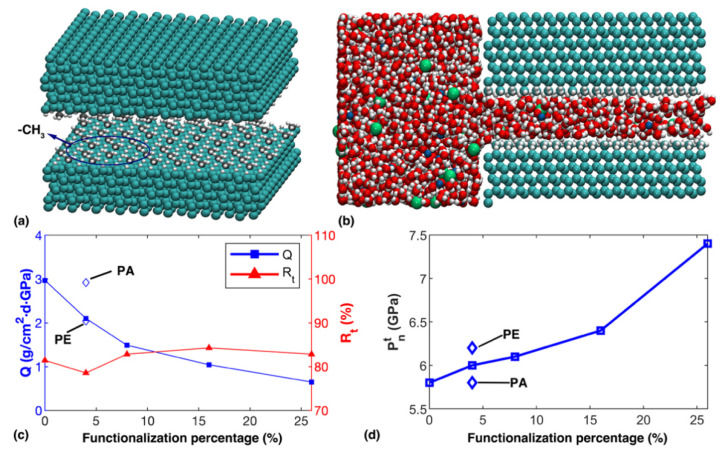
Transportation within the methyl-functionalized diamond nanochannel. (**a**) Schematic view of the methyl-functionalized diamond nanochannel; (**b**) Snapshot of solution transport within the nanochannel with 26% surface functionalization; (**c**) The water flux and ion retention rate as a function of the functionalization percentage; and (**d**) The transition nominal pressure as a function of the functionalization percentage. In (**c**,**d**), notation *PA* and *PE* represent the parallel and perpendicular alignment, respectively.

Before concluding, we also investigated how the permeability of the nanochannel could be tuned by surface functionalization. For illustration, we adopted methyl groups (-CH_3_) to replace hydrogen atoms. The hydrogen atoms were randomly selected by our in-house code, and functionalization for adjacent C atoms were avoided to maintain a low energy configuration, and the functionalization percentage was defined as the ratio between the quantity of methyl groups and the original quantity of hydrogen atoms in the diamond nanochannel. Before functionalization, the channel height was 10.2 Å. The model information is summarized in Supplementary Material Table S3. As shown in Figure 8a, -CH_3_ functional groups broke the smoothness of the interior surface of the nanochannel.

Due to the presence of functional groups, layered water molecules were not observed in the nanochannel (Figure 8b). The resulting disturbance from the functional groups was found to cause obvious reduction in terms of water flux (Figure 8c), which is consistent with previous observations for nanochannels with smaller height. Despite that, the ion retention rate was found to fluctuate around 80%, without clear correlation with surface functionalization. Further, the transition nominal pressure evidently increased when the functionalization percentage increased (Figure 8d). For example, $P_{n}^{t}$ at 26% functionalization (~7.5 GPa) was about 25% higher than that of the pristine channel. Note that two samples, with the same 26% functionalization but different random patterns were examined, from which totally the same water flux and ion retention rates were estimated.

Besides random functionalization, it was possible to prepare specific functionalization patterns in experiments, which was expected to affect the permeability of the nanochannel. Here, two additional simulations with 4% functionalization were carried out, where the functional groups aligned parallel (*x*-axis) and perpendicular (*y*-axis) to the transportation direction, respectively. Compared with the channel with randomly distributed functional groups, the parallel alignment increased the water flux (Q≈2.92 g/cm^2^·d·GPa) as the disturbance induced by the functional groups was minimized. In comparison, regarding the perpendicular alignment there was slight decrease in the water flux (Q≈2.04 g/cm^2^·d·GPa). The ion retention rate was nearly unchanged (~78%), while the transition nominal pressure shared the same changing tendency with the water flow but the difference was marginal, compared with that of the random counterpart (Figure 8d).

## 4. Conclusions

Based on atomistic simulations, this work investigated the permeability of hexagonal diamond nanochannels for NaCl solution. Compared with the multilayer graphene nanochannel, with the same channel height, the diamond nanochannel exhibited stronger permeability with an 84% increase in water flux, while ensuring an 88% ion retention rate. The whole transportation process can be divided into three stages: the diffusion stage, the transition stage and the flow stage. Increasing the channel height was found to improve the water permeability of the diamond nanochannel with increased water flux but reduced ion retention rate. In comparison, the channel length exerted ignorable influence on the water permeability of the channel. Further simulations revealed that temperatures below room temperature (~300 K), or approaching boiling point, exerted insignificant impact on the permeability of the nanochannel. Between 300 and 350 K, a remarkable increase in water flux was observed, together with a significant reduction in ion retention rate. Increasing solution concentration was found to exert ignorable influence on water permeability of the channel but reduced ion retention capacity and increased transition nominal pressure. Additional investigations showed that the permeability of the nanochannel can be suppressed by surface functionalization within the channel. Overall, this work provides a comprehensive atomic insight into the transportation process of NaCl solution in a diamond nanochannel, and the established understanding could be beneficial for the design of advanced nanofluidic devices.

## Data Availability

The data that support the findings of this study are available from the corresponding authors on reasonable request.

## Supplementary Materials

The following supporting information can be downloaded at: https://www.mdpi.com/article/10.3390/nano12111785/s1, Figure S1: Water flow (per unit time per unit area) as a function of pressure for the multilayer graphene and diamond nanochannel with a height of 6.8 Å. The gradient of the profile represents the water flux. It is seen that the water flow increases almost linearly when pressure exceeds 10 GPa, suggesting a stable water flux within the nanochannel. Obviously, diamond nanochannel has a larger water flux compared with the graphene nanochannel; Figure S2: Potential energy change ($∆E^{PE}$) and displacement (*D*) of water molecules as a function of pressure for the diamond nanochannel with a height of 10.2 Å. Crossing between the two linear fitting lines identify the transition pressure when flow stage occurs; Figure S3: Trajectory of a selected water molecule in a diamond nanochannel with a height of 10.2 Å; Table S1:Parameters of the Lennard-Jones potential; Table S2: Summary of models with different channel heights, lengths, temperatures and solution concentrations; Table S3: Summary of models with functional groups; Video S1: Transportation of 1M NaCl solution in a multilayer graphene nanochannel with a height of 6.8 Å; Video S2: Transportation of 1M NaCl solution in a diamond nanochannel with a height of 6.8 Å; Video S3: Transportation of 1M NaCl solution in a diamond nanochannel with a height of 12 Å. Ref. [42] is cited in Supplementary Matreials.
