# Supplementary material for: Atomistic Simulations of the Permeability and Dynamic Transportation Characteristics of Diamond Nanochannels
Source: Nanomaterials (Basel). 2022 May 24;12(11):1785. doi: 10.3390/nano12111785 (PMC9181998; doi:10.3390/nano12111785)
Supplement: Supplementary file 1 [file nanomaterials-12-01785-s001.zip › Supplementary Material.pdf]

nanomaterials

Supplementary Material

Atomistic Simulations of the Permeability and Dynamic Transportation Characteristics of Diamond Nanochannels

Bingqing Li [1], Bin Dong [1], Tianxiang Shi [1], Haifei Zhan [1,2,3,*] and Yongqiang Zhang [1,*]

[1] College of Civil Engineering and Architecture, Zhejiang University, Hangzhou 310058, China; 21912097@zju.edu.cn (B.L.); 22012088@zju.edu.cn (B.D.); stxzj@zju.edu.cn (T.S.)
[2] School of Mechanical, Medical and Process Engineering, Queensland University of Technology (QUT), Brisbane QLD 4001, Australia
[3] Center for Materials Science, Queensland University of Technology (QUT), Brisbane QLD 4001, Australia
* Correspondence: zhan_haifei@zju.edu.cn (H.Z.); cyqzhang@zju.edu.cn (Y.Z.)

Table S1. Parameters of the Lennard-Jones potential [1].

Atom type	σ (Å)	ε (kcal/mol)
H_W-H_W	0	0
O-O	3.166	0.1554
Na-Na	2.160	0.3526
Cl-Cl	4.831	0.0128
C-H_W	2.690	0.0383
C-O	3.436	0.0850
C-Na	2.775	0.1562
C-Cl	4.111	0.0298
H_D-H_W	1.990	0.0212
H_D-O	2.578	0.0574
H_D-Na	2.075	0.0865
H_D-Cl	3.411	0.0165
O-Na	2.663	0.2340
O-Cl	3.999	0.0446
H_W-Na	2.075	0.0865
H_W-Cl	3.411	0.0165
Na-Cl	3.496	0.0672

H_D: hydrogen atom in the diamond structure, H_W: hydrogen atom in water

Table S2. Summary of models with different channel heights, lengths, temperatures and solution concentrations.

Cases	Channel height (Å)	Channel length (Å)	Temperature (K)	NaCl concentration (M)	Simulation time (ps)
1	5	40	300	1	1300
2	6.8	40	300	1	1300
3	8	40	300	1	1300
4	9	40	300	1	1300
5	10.2	40	300	1	1300
6	12	40	300	1	1300
7	10.2	30	300	1	1300
8	10.2	40	300	1	1300
9	10.2	50	300	1	1300
10	10.2	60	300	1	1300
11	10.2	40	275	1	1300
12	10.2	40	290	1	1300
13	10.2	40	300	1	1300
14	10.2	40	310	1	1300
15	10.2	40	330	1	1300
16	10.2	40	350	1	1300

17	10.2	40	370	1	1300
18	10.2	40	390	1	1300
19	6.8	40	300	0.5	1300
20	6.8	40	300	1	1300
21	6.8	40	300	1.5	1300
22	6.8	40	300	2	1300

Table S3. Summary of models with functional groups.

Cases	Methyl contents (%)	Channel Height (Å)	Channel length (Å)	Temperatures (K)	NaCl concentration (M)	Simulation times (ps)
1	26	10.2	40	300	1	1300
2	26	10.2	40	300	1	1300
3	16	10.2	40	300	1	1300
4	8	10.2	40	300	1	1300
5	4	10.2	40	300	1	1300
6 (L)	4	10.2	40	300	1	1300
7 (W)	4	10.2	40	300	1	1300
8	0	10.2	40	300	1	1300

Note: Nanochannels for Cases 1-2 contain a same methyl content, but possess different random distribution patterns; L and W represent the methyl pattern parallel (x-axis) and perpendicular (y-axis) to transportation direction, respectively.

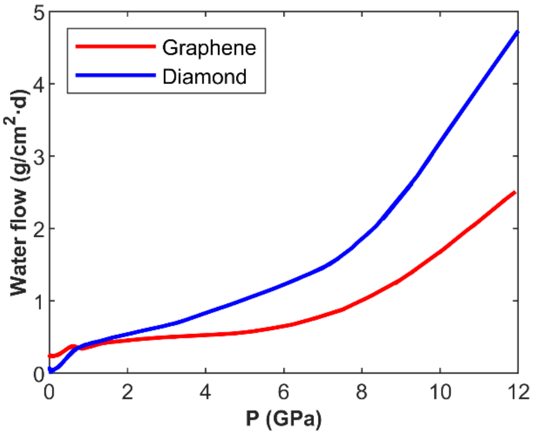

Figure S1. Water flow (per unit time per unit area) as a function of pressure for the multilayer graphene and diamond nanochannel with a height of 6.8 Å. The gradient of the profile represents the water flux. It is seen that the water flow increases almost linearly when pressure exceeds 10 GPa, suggesting a stable water flux within the nanochannel. Obviously, diamond nanochannel has a larger water flux compared with the graphene nanochannel.

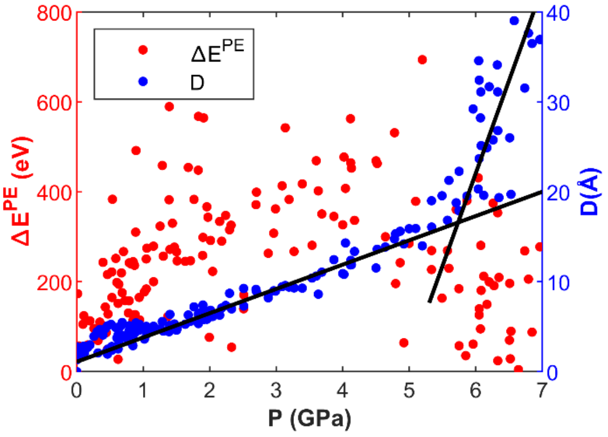

Figure S2. Potential energy change (ΔE^{PE}) and displacement (*D*) of water molecules as a function of pressure for the diamond nanochannel with a height of 10.2 Å. Crossing between the two linear fitting lines identify the transition pressure when flow stage occurs.

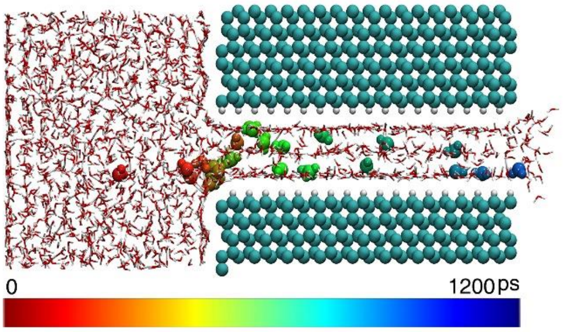

Figure S3. Trajectory of a selected water molecule in a diamond nanochannel with a height of 10.2Å.

Supporting Video.

Supporting Video S1: Transportation of 1M NaCl solution in a multilayer graphene nanochannel with a height of 6.8 Å;

Supporting Video S2: Transportation of 1M NaCl solution in a diamond nanochannel with a height of 6.8 Å;

Supporting Video S3: Transportation of 1M NaCl solution in a diamond nanochannel with a height of 12 Å;

References

1. Yu, Y.Z.; Fan, J.C.; Xia, J.; Zhu, Y.B.; Wu, H.A.; Wang, F.C. Dehydration impeding ionic conductance through two-dimensional angstrom-scale slits. Nanoscale 2019, 11, 8449-8457.
